# Cologne ergonomic measurement for robotic surgery (CEMRobSurg) using the Hugo™ RAS System

**DOI:** 10.1007/s00464-024-11129-7

**Published:** 2024-08-26

**Authors:** Stefanie Brunner, Dean Müller, Dolores T. Krauss, Rabi Raj Datta, Jennifer A. Eckhoff, Christian Storms, Benedikt von Reis, Seung-Hun Chon, Thomas Schmidt, Christiane J. Bruns, Hans F. Fuchs

**Affiliations:** 1 Department of General, Visceral, Cancer and Transplant Surgery, University Hospital of Cologne, 50937 Cologne, Germany; 2 Faculty of Medicine, University of Cologne, 50923 Cologne, Germany; 3 Medical University of Białystok, ul. Kilinskiego 1, 15-089 Białystok, Poland; 4 Surgical Artificial Intelligence and Innovation Laboratory, Massachusetts General Hospital, 55 Fruit Street, Wang C3-339, Boston, USA

**Keywords:** Ergonomics, Robotic surgery, Hugo™ RAS System, Heart rate variability, Posture

## Abstract

**Background:**

The ergonomic advantages and potential challenges that robotic surgery poses to the well-being of surgeons are mainly unexplored. The most recent surgical robot introduced on the European market is the Hugo™ RAS System by Medtronic. This study aims to evaluate the ergonomic benefits of the Hugo™ RAS System, which is available in our training laboratory, CeMIT (Center for Medical Innovation and Technology Cologne).

**Methods and procedures:**

Using the previously established Cologne Ergonomic Measurement Setup for Robotic Surgery (CEMRobSurg), we measured three parameters related to ergonomic posture from subjects with different levels of surgical expertise (laypeople, medical students, surgical residents, and expert robotic surgeons). The heart rate was measured continuously using a polar band. The noise level was measured while using the Hugo™ RAS System, and automated photographs using our locally developed methodology were captured of the participant every 2 s to assess body posture. The ergonomic measurements were conducted while the subject performed the same standardized robotic training exercises (Peg Board, Rope Walk, and Ring Walk).

**Results:**

A total of 53 participants were enrolled in this study. The average noise level during all measurements was 54.87 dB. The highest stress level was measured in surgical residents with a sympathetic nervous system index (SNS index) of 1.15 (min − 1.43, max 3.56). The lowest stress level was measured in robotic experts with an SNS index of 0.23 (min − 0.18, max 0.91). We observed a risk-prone positioning of the neck and elbow in medical students (mean 39.6° and 129.48°, respectively). Robotic experts showed a risk positioning in the knee and hip region (mean 107.89° and 90.31°, respectively).

**Conclusion:**

This is the first study to analyze and objectify the ergonomic posture of medical students, surgical trainees, surgeons, and laypeople using the open console, modular Hugo™ RAS System. Our findings offer recommendations for operating surgeons and allow for a comparative analysis between the different robotic systems. Further evaluations in real-time operative scenarios will follow.

In the last decade, robotic innovations have dominated the evolution from open procedures to minimally invasive surgery [1]. Around the world, robotic surgery is rapidly increasing. In 2020, its global volume was 1.24 million robotic procedures, with a dominance in the USA, accounting for 70.6% [2]. Data on the use of robotic surgery in low- and middle-income countries are limited. The evolution of surgical robotic systems began in the 1970s in the USA. The National Aeronautics and Space Administration (NASA) became a pioneer in the development of surgical robots. The first visceral robotic surgery, a cholecystectomy, was performed in 1998 using the Da Vinci Surgical System (Intuitive surgical, Inc., Sunnyvale, CA, USA). A continuously increasing number of surgical subspecialties included robotic surgery in their routine practice [3]. Robotic surgery demonstrated improved outcomes and an increased quality of life in patients [4]. In oncologic surgery, first studies demonstrate equivalent outcomes of robotic-assisted procedures compared to open and laparoscopic procedures [5–7].

Due to unparalleled high-resolution, three-dimensional visual qualities and an integrated surgeon-controlled camera, robotic systems offer a more steady and thorough overview of the operative field. Additionally, the console filters out slight tremors to allow for a more precise preparation. Robotic instruments, exhibiting seven degrees of freedom alongside improved triangulation and offer better access to anatomically challenging regions [8]. In summary, robotic surgery offers an enhanced range of motion, better dexterity, more precise instrument control, and superb visualization. Unsurprisingly these enticing qualities have led to the introduction of a wide variety of robotic systems to the market [9, 10], besides the Da Vinci surgical system (Intuitive, Sunnyvale, CA, USA).

The Hugo™ RAS System (Medtronic, Dublin, Republic of Ireland©) obtained the CE (Conformité Européenne) mark for adult gynecological, urological, and general procedures in early 2022 [11]. The system consists of a console and a system tower (Fig. 1). In contrast to the Da Vinci, the Hugo™ RAS features four modular arm carts, for individualized surgical setups and approaches [12] and an open console that facilitates communication among the operating team.

**Fig. 1:**
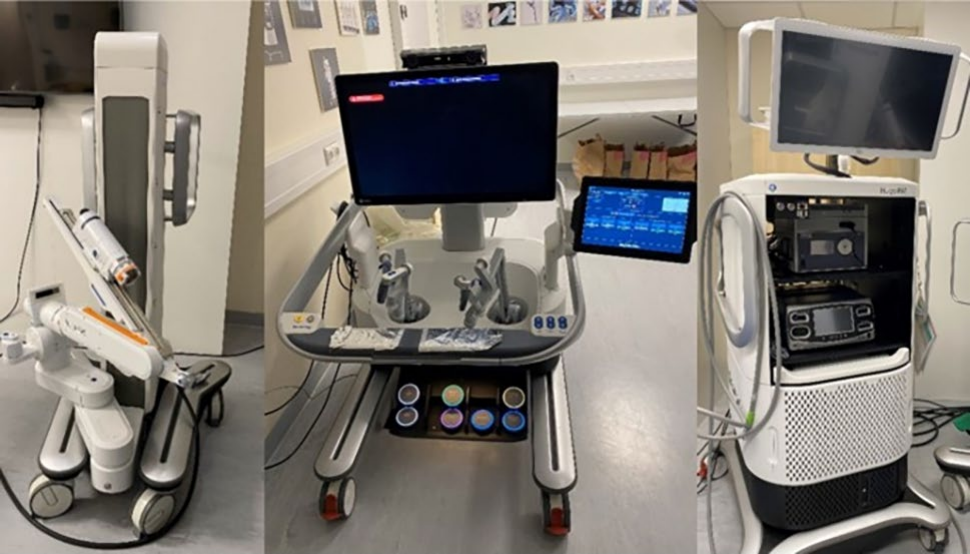
Setup of the Hugo™ RAS System consisting of arm cards, console, and a system tower

The introduction of the Hugo™ RAS System aimed to provide an alternative robotic platform with a potentially more ergonomic and personalized working environment [13]. The operating surgeon can individualize ergonomic settings by adjusting the armrest height, foot pedal, and monitoring height. During instrument rotation, the Hugo™ RAS System allows the surgeon to return his wrist to a neutral position before further moving the instrument. The system also includes advanced safety features, such as the activation of controllers as the surgeon looks at the monitor [14]. In general surgery, the system is licensed for colorectal procedures, cholecystectomies, appendectomies, and hernia repair [15, 16].

Over the last years ergonomics, the scientific exploration of the interaction between humans and their respective surroundings, has attained increased importance in the surgical workspace [17]. The field is classified in organizational, cognitive components and physical ergonomics, with the latter focusing on musculoskeletal discomfort [18].

Due to repetitive movements and static and awkward postures, surgeons have a significantly elevated risk for musculoskeletal pain and long-term health consequences, compared to the normal population [19]. Epstein et al. described a 60% prevalence for the development of neck pain and 52% for shoulder pain among surgeons. Moreover, the elevated prevalence of degenerative lumbar spine disease of 19% and rotor cuff pathologies of 18% is forcing many surgeons to early retirement. Robotic surgery aims to provide surgeons with favorable workplace ergonomics compared to conventional open or laparoscopic surgery [20, 21]. Rather than standing at the operating table, the surgeon is seated at a console, which offers individually adjustable armrests and foot pedals for decreased strain on the extremities. However, the ergonomic benefits and potential challenges of robotic systems should be carefully evaluated and compared. To date, most scientific explorations around surgeon ergonomics focus on the Da Vinci surgical system, featuring a closed, microscope-like surgeon console [22]. In contrast, this study presents an evaluation of the ergonomic features offered by the new Hugo™ RAS System, an open surgeon console, modular system, using a standardized ergonomic measurement setup in a dry lab setting.

## Methods

### Study participants

We conducted ergonomic measurements according to the Cologne Ergonomic Measurement Setup for Robotic Surgery (CEMRobSurg) on four subject groups with varying levels of surgical expertise: laypeople, medical students, surgical residents, and experienced robotic surgeons. Laypeople had no prior exposure to surgery, whereas medical students were enlisted in the clinical track of their studies (6th to 12th semester). The group of surgical residents exhibited experience in bedside assistance but no console training. Board-certified surgeons experienced in robotic-assisted minimally invasive procedures were included. All surgeons were affiliated with the Department for General, Visceral, Cancer and Transplant Surgery, Faculty of Medicine of the University Hospital Cologne. Finally, laypeople were asked to participate. Besides training status and surgical expertise, the following demographic information was collected from the participants: age, gender, handedness, gaming experience, laparoscopic and robotic experience, and surgical experience. Additionally, the participants were asked, how many minutes per day they were gaming during their respective time of actively gaming.

#### Study design

The objective of this prospective, single-center study was to identify ergonomic challenges and objectify subject stress levels when using the Hugo™ RAS System.

**Fig. 2:**
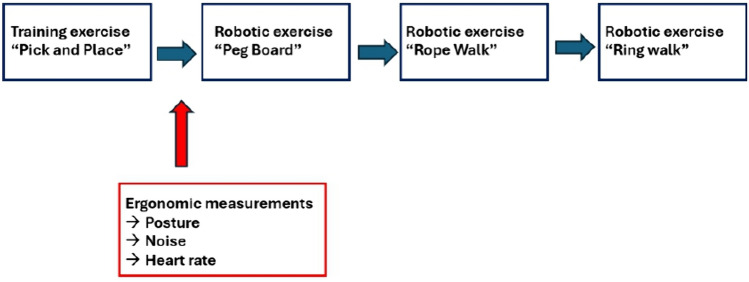
Visual timeline of tasks and measurements during the data collection process

Each participant completed a series of structured and validated training exercises while the ergonomic measurements were conducted. The measurements were performed at the training laboratory CeMIT at the University Hospital of Cologne. A visual timeline depicting the sequence of tasks is demonstrated in Fig. 2.

At the beginning, all participants received a structured and standardized introduction. Teaching included the “buttonology” of the robot, including basic handling of the robotic console and controller. All instructors were specifically trained in handling the Hugo™ RAS System by representatives of Medtronic. The participants were asked to perform the “Pick and Place” exercise from Mimic Technologies (Seattle, USA), where colored objects are placed in matching colored containers. This exercise was repeated three times and served to familiarize the robotic console. No ergonomic measurement was performed during this first robotic simulation.

**Fig. 3:**
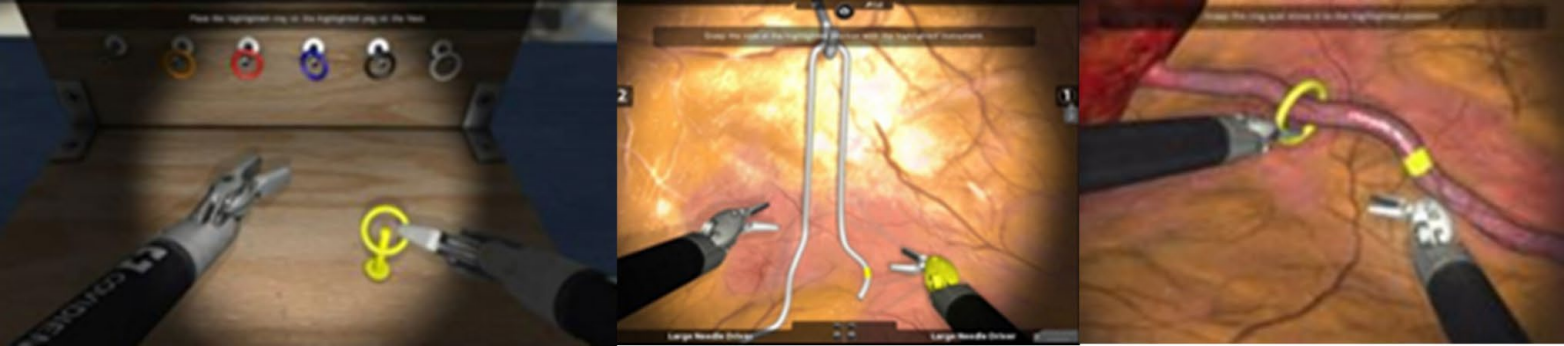
Robotic exercises: Peg Board, Rope Walk, and Ring Walk by Mimic Technologies

The beginning of each trial was defined as the moment when the participant first unlocked the robotic handles. The end of each trial was defined as the moment when the participant completed the task, and the system automatically ended the surgical simulation. The participants performed three robotic exercises: Peg Board, Rope Walk, and Ring Walk (see Fig. 3). All exercises were constructed by Mimic Technologies (Seattle, USA). Each robotic task was completed three times. With the Peg Board task, participants were asked to pick up and transfer rings from the peg board to a single peg on the floor. The participants learned to use both instruments with the task Rope Walk. Both instruments selectively grasp marked areas of a flexible string. In the last simulation, Ring Walk, the participants learned to switch between camera and instrument control effectively. Based on our previously published study protocol, the exercises were chosen where laparoscopic skills were monitored [23]. These exercises were chosen based on their successful deployment as valid measurement tools in previous explorations [24].

#### Ergonomic measurements

The Cologne Ergonomic Measurement for Robotic Surgery (CEMRobSurg) setup was developed in our Center and previously used to evaluate ergonomics in esophageal cancer surgery (ERASE study; German Clinical Trials Register (DRKS00025022) using the Da Vinci Surgical System.

#### Evaluation of posture

**Fig. 4:**
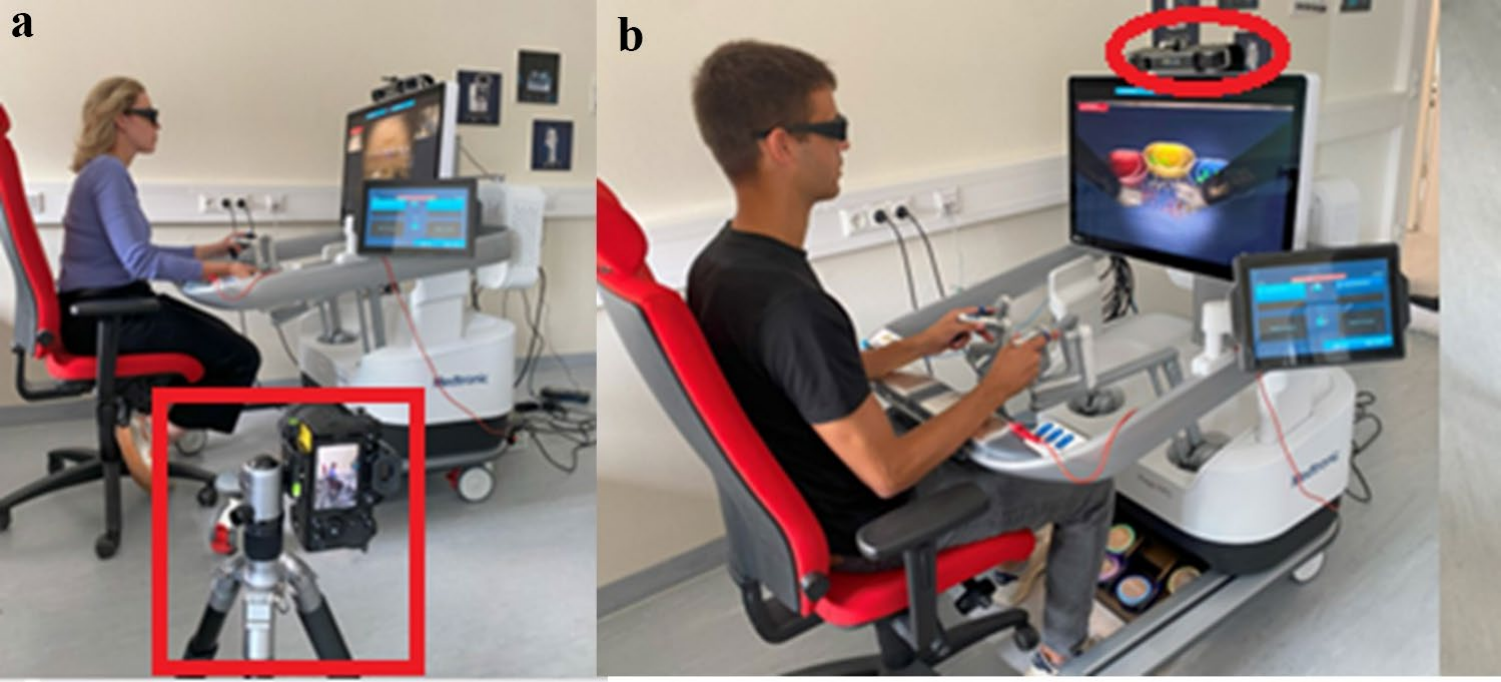
**a** Camera usage to evaluate the participant’s posture. **b** A sound recorder was used to assess the general noise in the room

**Fig. 5:**
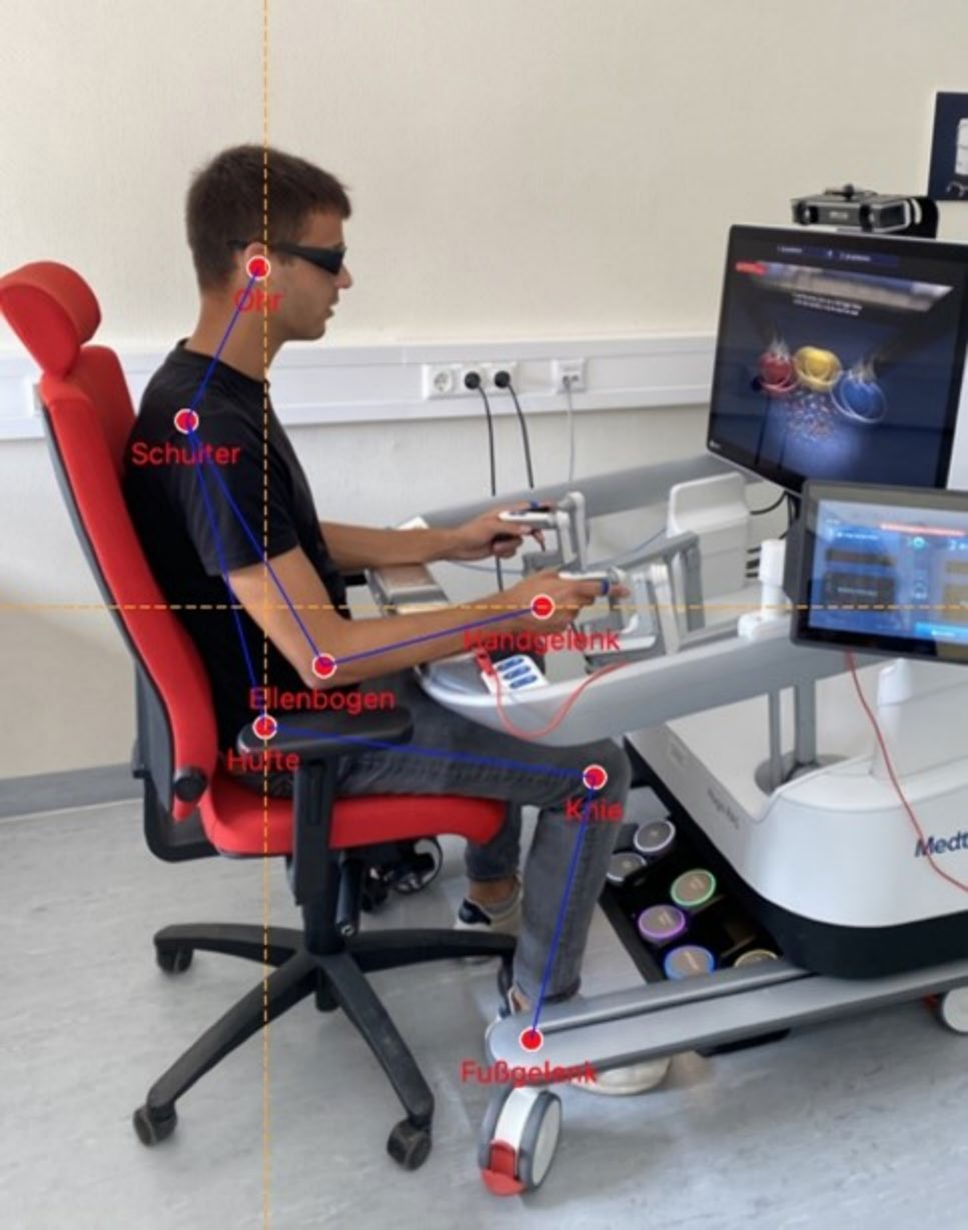
Evaluation of the body posture. All joint angles are calculated to determine the ergonomic body posture using the Hugo™ RAS System

To evaluate the posture of all participants, a video camera was installed at a 90° angle toward the Hugo™ RAS System. Before the participant started the simulation, the instructor ensured that the video camera detected the entire body, especially the armrest. As soon as the simulation began, the camera took a picture every 2 s (Fig. 4a). The pictures were sorted into “console time” and “non-console time.” The non-console time was defined as situations where the participant was not actively participating in the simulation exercise. Only the console time was further analyzed. Afterward, the pictures were sorted into 5-min sections. For every period, a merged picture was fabricated using a specially generated program in Python (Python Software Foundation, Delaware, USA). To calculate angles, axes are placed through the participant’s joints (Fig. 5). Angles of the neck, shoulder, torso, elbow, hip, knee, and forearm region were evaluated. The individual axes were then compared with angles defined as ergonomically low risk [25].

To assess the surgeon’s posture, an AI model by OpenPose was used. OpenPose is a machine learning model that estimates body pose in an image. With the coordinates of all joints estimated, the angles of each individual joint can be calculated using trigonometry. To achieve this, vectors were created from each of the adjacent joints to the joint being assessed. The angle between two vectors then can be calculated using the inverse trigonometric function of the cosine (arccos):

$$\Theta =\text{arccos} \left(\frac{{x}_{a}{x}_{b}+{y}_{a}{y}_{b}}{\sqrt{{{x}_{a}}^{2}+{{y}_{a}}^{2}} \cdot \sqrt{{{x}_{b}}^{2}+{{y}_{b}}^{2}}} \right),$$

where vector *a* and *b* are the vectors, connecting adjacent joints to the evaluated joint.

Using artificial intelligence to evaluate the landmarks on the human body makes assessment and evaluation much faster, even allowing real-time analysis of posture.

For optimal ergonomic positioning, the feet should rest on the ground. The optimal angle for the knees and elbows is set at ≥ 90°. The forearm should be positioned parallel to the ground floor on the armrest. An angle between 110° and 120° is recommended for the hip region. With focus on the neck position, a 10°–15° angle is advocated for sitting procedures. It is recommended that the torso be rested at 0°–10°. These angles are in accommodation with the recommendations for occupational safety while using a workstation with a monitor [26, 27].

#### Sound level measurement

A sound level measurement was performed to objectively evaluate the noise nuisance while performing simulation exercises on the Hugo™ RAS System. Therefore, the PCE-322A device (PCE Deutschland GmbH, Germany) is used. The sensor is positioned close to the respected participant (Fig. 4b). Noise levels are measured in decibels (dBA).

#### Heart rate and stress index

The participant’s stress level while performing the simulation was detected by measuring heart rate (HR). Every participant wore an ECG chest strap and corresponding sensor during the measurement. The data were analyzed using the Kubios software (Kubios Oy, Kuopio, Finland).

The mean HR was calculated to objectify the state of excitation. An increase in HR represents a high state of stress since the sympathetic nervous system is activated [28]. The parasympathetic nervous system index (PNS Index) and the sympathetic nervous system index (SNS Index) are also analyzed. These parameters are used to evaluate the objective stress level. An increased SNS index alludes to a higher stress level. In reverse, a lower PNS index indicates a higher stress level [29].

The LF/HF ratio describes the balance between sympathetic and parasympathetic influences. The higher the LF/HF Ratio, the higher the sympathetic tone and the higher the stress level [30].

### Statistical analysis

The collected data were entered in Excel (Version 14.0.7229.5, Microsoft Corporation, Redmond, WA, USA) and analyzed using SPSS Statistics 24 (IBM Corporation, Armonk, NY, USA) through descriptive statistical analyses. All continuous variables were expressed as median and range; all categorical variables were expressed as sum and percentage. The size and composition of the study groups were chosen based on the clinical routine. Since the study cohort is *n* = 53 and the respected subgroups are small, a statistical analysis including confidence intervals or *p* values are not recommended.

## Results

### Study cohort

The study cohort (*n* = 53) consists of 30 medical students, 14 surgical residents, four experts in robotic surgery, and five laypeople. The participants’ characteristics are summarized in Tables 1 and 2. The vast majority of the participants were right handed. Around 40% of the participants were wearing glasses.

**Table 1:** Characteristics of the study cohort with regard to gaming experience

	Medical students	Residents	Robotic experts	Laypeople
Age (years)	26.17 (min 22, max 33)	29.86 (min 26, max 33)	42.25 (min 37, max 48)	43.8 (min 18/max 70)
Gender (male)	56.67%	42.86%	100%	40%
Handedness (right)	76.67%	85.71%	75%	100%
Glasses (yes)	43.33%	35.71%	25%	60%
Owning a gaming console (yes)	33.33%	35.71%	25%	0%
Years of gaming	7.23 (min 0, max 27)	4.5 (min 0, max 15)	3.5 (min 0, max 5)	3.4 (min 2/max 10)
Minutes of gaming/day	37.5 (min 0, max 180)	112.5 (min 0, max 480)	20 (min 0, max 30)	32 (min 10/max 120)

*N/A* not applicable

**Table 2:** Characteristics of the study cohort with regard to robotic and laparoscopic experience

	Medical students	Residents	Robotic experts
Semester	8 (min 6, max 12)	N/A	N/A
Laparoscopic exposure	10%	100%	100%
Robotic experience	0%	50% none, 50% simulation	5.5 (min 2, max 14)
Interest in robotic surgery	70%	100%	100%
Year of surgical training	N/A	3.57 (min 1, max 8)	14.75 (min 10, max 23)
Surgical experience	N/A	50% low, 42.86% middle, 7.14% high	100% high

*N/A* not applicable

Concerning the gaming experience, 33.33% of the medical students and 35.71% of the surgical residents were in possession of a gaming console. However, none of the laypeople owned a gaming console. However, laypeople were gaming 3.4 years during their active years of gaming. The medical students had the most gaming experience, averaging 7.23 years per participant. The surgical residents were gaming 112.5 min per day during their active years of gaming—considerably longer than any other group.

Only 10% of the medical students were previously exposed to laparoscopic procedures. Half of the surgical residents were in contact with robotic simulation. The robotic experts had an average of 5.5 years of robotic experience.

#### Sound level measurement

The average noise level during all measurements was 54.87 dB. The maximal noise level was recorded at 87.3 dB, and the minimum noise level was 40.5 dB.

#### Heart rate and objective stress level

All parameters are summarized in Table 3. A higher heart rate (HR) suggests an increased sympathetic activation or physical stress and, therefore, indicates an elevated stress level among the study participants. The highest mean HR was observed in surgical residents with 81.25 bpm (min 53, max 99). The lowest mean HR was measured in robotic experts with 67 bpm (min 62, max 76). An HR of 80.09 bpm (min 64, max 119) was calculated among medical students. Laypeople showed an HR of 69.25 (min 62, max 75).

**Table 3:** Heart rate variability (HRV) measurement and objective stress parameters

	Medical students	Residents	Robotic experts	Laypeople
Mean RR (ms)	766.55 (min 636, max 893)	759.17 (min 607, max 1133)	899.75 (min 789, max 949)	870 (min 800, max 966)
Mean HR (bpm)	80.09 (min 64, max 119)	81.25 (min 53, max 99)	67 (min 63, max 76)	69.25 (min 62, max 75)
Min HR (bpm)	64.64 (min 55, max 94)	65.75 (min 57, max 84)	57.25 (min 54, max 62)	59.5 (min 51, max 65)
Max HR (bpm)	106.68 (min 85, max 116)	104.42 (min 84, max 115)	75.5 (min 54, max 93)	91.75 (min 71, max 106)
Stress Index	9.35 (min 6.2, max 18.6)	10.09 (min 5.8, max 19.1)	10 (min 9.1, max 11.5)	10.8 (min 4.7, max 19.6)
LF/HF Radio	3.66 (min 1.41, max 7.1)	3.09 (min 1.07, max 6.23)	6.75 (min 2.41, max 17.86)	2.03 (min 0.87, max 3.43)
PNS Index	− 1.04 (min − 3.05, max 0.3	− 0.94 (min − 2.26, max 1.88)	− 0.49 (min − 1.25, max − 0.02)	− 0.04 (min − 1.3, max 2.15)
SNS Index	1 (min − 0.37, max 5.39)	1.15 (min − 1.43, max 3.56)	0.23 (min − 0.18, max 0.91)	0.42 (min − 1.09, max 1.55)

*RR* Riva-Rocci, *ms* millisecond, *HR* heart rate, *bpm* beats per minute, *LF* low frequency, *HF* high frequency, *PNS* parasympathetic nervous system, *SNS* sympathetic nervous system

The stress index was analyzed among all study groups. The highest stress level was measured in surgical residents with 10.09 (min 5.8, max 19.1). Medical students showed the lowest stress index with 9.35 (min 6.2, max 18.6). A stress level of 10 (min 9.1, max 11.5) was measured among robotic experts. Laypeople showed a stress level of 4.7, max 19.6).

Furthermore, the PNS (parasympathetic nervous system) index and SNS index were analyzed. The PNS index represents the parasympathetic nervous system. Lower PNS index values are expected during stress, reflecting decreased parasympathetic activity. Concerning the study cohort, medical students showed the lowest PNS index with an average of − 1.04 (min − 3.05, max 0.3), indicating a substantial level of stress. The highest PNS index was measured among laypeople with an average of − 0.04 (min − 1.3, max 2.15). The residents showed an average PNS index of − 0.94 (min − 2.26, max 1.88). Robotic experts showed a PNS index − 0.49 (min − 1.25, max − 0.02).

**Fig. 6:**
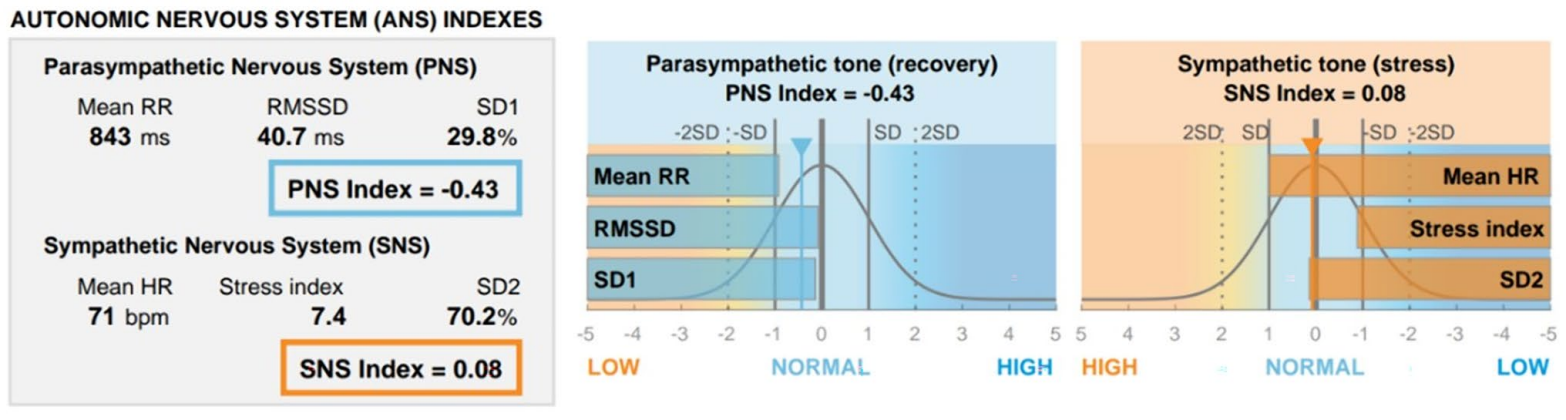
Evaluation of the objective stress levels of Student Nr. 1

The SNS index value represents the activity of the sympathetic nervous system. The higher the SNS Index, the higher the stress level of the individual participant. The measurement of the objective stress level of student nr. 1 is exemplary, presented in Fig. 6. The highest stress level was measured in surgical residents with an SNS index of 1.15 (min − 1.43, max 3.56). The lowest stress level was measured in robotic experts with an SNS index of 0.23 (min − 0.18, max 0.91). Medical students showed a SNS index 1 (min − 0.37, max 5.39). Among laypeople, an SNS index of 0.42 (min − 1.09, max 1.55) was measured.

The highest LF/HF Ratio was measured in robotic experts with 6.75 (min 2.41, max 17.86). The lowest LF/HF Ratio was detected among laypeople with 2.03 (min 0.87, max 3.43). Medical students showed a LF/HF Ratio 3.66 (min 1.41, max 7.1). Surgical residents showed a LF/HF Ratio 3.09 (min 1.071, max 6.23).

#### Evaluation of posture

To evaluate the participants' posture, the following joints and angles were analyzed: knee, hip, shoulder, neck, elbow, torso, and forearm. The average angles in which the participant remained during the exercises are summarized in Table 4.

**Table 4:** Measurement of the participants postures, all values are presented as angles

	Medical students	Residents	Robotic experts	Laypeople
Knee	81.21 (min 74.63, max 87.32)	86.82 (min 84.35, max 89.29)	107.89 (min 90.78, max 124.99)	84.66 (min 66.35, max 102.96)
Hip	113.37 (min 90.66, max 136.09)	94.07 (min 93.57, max 94.58)	90.31 (min 83.28, max 97.34)	102.67 (min 90.55, max 114.79)
Shoulder	27.83 (min 18.54, max 37.11)	25.4 (min 18.91, max 31.88)	33.94 (min 29.36, max 38.51)	32.51 (min 30.43, max 34.60)
Neck	39.96 (min 26.4, max 53.53)	16.03 (min 7.57, max 24.5)	26.29 (min 24.85, max 27.74)	29.38 (min 10.25, max 48.51)
Elbow	129.48 (min 124.19, max 134.77)	111.3 (min 106.3, max 116.3)	114.79 (min 105.55, max 124.03)	125.15 (min 112.42, max 137.89)
Torso	15.92 (min 0.77, max 31.07)	0.91 (min 0.8, max 1.02)	6.139 (min 2.12, max 10.15)	10.047 (min 4.05, max 16.04)
Forearm	3.498 (min 2.15, max 4.84)	4.21 (min 1.82, max 6.60)	8.679 (min 5.66, max 11.69)	4.78 (min 1.42, max 8.13)

Surgical residents were seated in a nearly ideal knee posture of 86.82° (min 84.35, max 89.29). An angle of 90° in the knee and elbow is described to be beneficial. Robotic experts were seated with the most insufficient knee posture compared to the other study groups with 107.89° (min 90.78, max 124.99). Medical students showed an average knee posture of 81.21° (min 74.63, max 87.32). Laypeople were seated with an average knee angle of 84.66° (min 66.35, max 102.96).

In the hip region, medical students spent an average time at an angle of 113.37° (min 90.66, max 136.09). Recommended is an angle between 110° and 120°. Robotic experts showed a weak posture concerning the hip region of 90.31° (min 83.28, max 97.34). Surgical residents had an average hip posture of 94.07° (min 93.57, max 94.58). Laypeople had an average hip posture of 102.67° (min, 90.55, max 114.79°).

With a focus on the neck position, medical students showed an average of 39.96° (min 26.4, max 53.53). It is recommended to sit at a workstation at an angle of 10°–15°. Surgical residents held the most ideal neck position with 16.03° (min 7.57, max 24.5).

The torso should be rested in a 0°–10° position while seated at a workstation with a monitor. The ideal position was detected among the surgical residents with an average position of 0.91° (min 0.8, max 1.02). Medical students, however, were seated at a 15.92° (min 0.77, max 31.07) angle – which represented the worst seating angle of the torso among all groups.

Concerning the elbow region, medical students showed the most improvable position in the elbow region of 129.48° (min 124.19, max 134.77). The ideal elbow position was monitored among the surgical residents at 11.3 (min 106.3, max 116.3).

## Discussion

This study offers the first quantifiable evaluation of surgeon ergonomics with the Hugo™ RAS System. Alongside an increasing number of robotic procedures across all surgical disciplines, objectifying and improving surgeon ergonomics is of great interest to the surgical community [31].

This study was performed in a controlled training environment in the CeMIT at the University Hospital of Cologne focusing entirely on the Hugo™ RAS System and evaluating a carefully selected study cohort with varying degrees of robotic surgical expertise. Besides experienced robotic surgeons, we evaluated ergonomic parameters in medical students, surgical residents, and laypeople. Besides offering a comprehensive analysis of ergonomics and postures related to musculoskeletal conditions and workplace comfort, this study offers comparative insights into the handling of robotic systems by laypeople, such as medical students and complete laypeople. Moreover, this study analyzes the ergonomics of surgical residents, with prior exposure to laparoscopic surgery but at the relative beginning of their robotic surgical training, allowing for insights into the evolvement of posture throughout surgical training.

To evaluate the ergonomic performance, all participants performed numerous simulation exercises. The selection of the exercises was based on previously published studies that evaluated suitable training exercises for laparoscopic surgeries. We obligated ourselves to a standardized measurement for all study participants. Especially the automated documentation of the participants’ postures using a photography and AI-based recognition has not yet been described.

Our study group specifically investigated the gaming experience of the participants. It is interesting to detect that medical students had an average gaming experience of 7.23 years and surgical residents spent an average of 112.5 min per day gaming during their active phase of video gaming. These study groups invested more time in video gaming than robotic experts and laypeople. Several studies are available that correlate robotic simulation’s success to video gaming [32]. Robotic surgery has additional technical challenges, such as 3D viewing and operating without haptic feedback. All these challenges are addressed while playing video games. Harper et al. demonstrated that prior video game exposure could enhance robotic performance [33]. Our study examined the ergonomic positioning of the study cohorts, who were previously exposed to video gaming. In terms of ergonomic positioning during the simulations, surgical residents showed a nearly ideal knee posture of 86.82° and hip positioning of 113.37°.

As expected, robotic experts possessed the most experience in robotic surgery, with an average of 5.5 years. The knowledge and routine of the robotic surgeons reflects in a reduced perception of stress. The lowest HR was detected among the robotic experts at 67 bpm. Moreover, the SNS index was lower in robotic surgeons compared with the other study groups, indicating a low stress level [29]. This is in line with the current literature. Lefetz et al. identified relevant intraoperative stressors, such as inexperience with the robotic system and low quality of team communication [34].

Rodriguez et al. reported better ergonomic posture with growing robotic experience [35]. In contrast, our study demonstrated that even experienced robotic surgeons can improve their posture significantly while working on the Hugo™ RAS System, to prevent musculoskeletal issues in future. The knee and hip postures could be improved, especially in this study cohort.

The current ergonomic guidelines for robotic surgery are transferred from working at the microscope. Several studies are available that measure the ergonomic conditions and musculoskeletal burden of the surgeon while performing laparoscopic or robotic procedures. During laparoscopic surgery, the biceps, triceps, and deltoid regions are especially burdened compared to robotic procedures [36]. Our study found the neck to be the most stressed region while using the Hugo™ RAS System. This aligns with the current literature, indicating the neck and trunk to be the most burdened regions while performing robotic surgery [37]. However, robotic surgery proved to be ergonomically beneficial when compared to the respected laparoscopic procedure [36].

Additionally, noise pollution was measured during the simulation exercises. Even under laboratory-like conditions, the average noise level was measured at 54.87 dB. The maximal noise level was recorded at 87.3 dB. Several studies have shown the connection between noise pollution, stress, and the work efficiency of the operating team [38]. Studies showed that the noise level in the operating rooms exceeded the 30-dbA recommended threshold set by the World Health Organization [39]. Noise is responsible for adverse effects ranging from poor concentration to mental and physical stress [40]. An increase in noise pollution is measured, mainly when technologies are applied in the operating room. Consequently, noise-induced hearing loss is induced in the medical staff [41].

Our study has several limitations. The study was performed under laboratory-like conditions. When operating in real-life situations, the posture and stress level of the surgeon might be more challenged. Moreover, noise levels might even be heightened under real-life conditions. It has to be considered that HR measurement is influenced by several factors. The students and residents are younger in comparison to our laypeople and surgical experts which might influence the measurement. Further studies are needed to evaluate ergonomics in the operating field. Additionally, particular stressful and ergonomically challenging operating phases should be identified to sensitize the operating surgeon further to focus on their posture during challenging operating steps.

## Conclusion

This is the first study to evaluate the ergonomics of users with different expertise using the Hugo™ RAS System. With the open console design, new challenges and chances were detected. Since especially the neck in medical students and knee region in surgical experts are at risk, specific ergonomic training should be offered to the respected surgeons in order to reduce long-term health burden. Additionally, prevention of ergonomic challenges should be included in a robotic standardized curriculum. It will be interesting to compare the evaluated standards of our CEMRobSurg setup using the HUGO RAS System with other systems and in real-life OR scenarios.
